# The Role of Affective Bonds in a Specialized Psychosis Outpatient Clinic in Brazil

**DOI:** 10.1192/j.eurpsy.2026.12126

**Published:** 2026-07-31

**Authors:** A. F. C. Ximenes, L. V. D. Araujo, P. F. B. de Araújo, A. B. D. C. Alves, L. C. Carneiro, M. I. O. de Morais, F. O. Barbosa, B. D. A. A. L. T. de Melo, R. T. Carvalho, D. B. B. Ferreira, V. L. de-Melo-Neto, E. D. F. Correia

**Affiliations:** 1 Psychiatry, Universidade Federal de Alagoas; 2 Psychiatry, Universidade de Ciências da Saúde de Alagoas, Maceió; 3 Psychiatry, IMIP - Instituto de Medicina Integral Prof. Fernando Figueira, Recife, Brazil

## Abstract

**Introduction:**

Psychotic disorders pose significant challenges for treatment adherence, particularly in resource-limited settings. In Brazil’s public health system (SUS), many patients face precarious living conditions, food insecurity, and limited social support, while depending entirely on the SUS for access to antipsychotics. Beyond structural barriers, the therapeutic relationship may play a decisive role in adherence. Psychiatrist Nise da Silveira argued that the “line-life” connecting people with schizophrenia to reality can be sustained through affective bonds with therapists and caregivers (O Mundo das Imagens, Imagens do Inconsciente).

**Objectives:**

To explore experiential insights from a specialized psychosis outpatient clinic within the SUS, emphasizing the role of affective bonds in fostering adherence, continuity of care, and clinical stabilization.

**Methods:**

From March 2024 to February 2025, more than 100 patients with psychotic disorders were followed in a specialized outpatient clinic. Observational and reflective notes were collected on engagement, medication adherence, and therapeutic dynamics. Selected cases exemplify how affective connections between patients and clinicians influenced adherence, acceptance of treatment, and crisis management.

**Results:**

Adherence was frequently linked less to insight into illness or resource availability than to trust in clinicians. For instance, a patient with schizoaffective disorder, despite limited understanding of her condition, maintained adherence because of confidence in her physician. A patient with first-episode psychosis who had discontinued medication returned to the clinic and even agreed to hospitalization after developing trust with the medical team. Another patient with refractory schizophrenia regularly contacted his physician during crises to differentiate reality from delusion, underscoring the therapeutic role of affective reassurance. Despite limited socioeconomic resources, most patients achieved relative clinical stability, supported by continuous follow-up. Each patient is seen by the same physician throughout the year-long rotation, which strengthens therapeutic bonds, and some maintain contact with former doctors after transition, reflecting the enduring impact of these connections. Overall, affective engagement emerged as a key factor in adherence, reducing relapses, hospital readmissions, and treatment abandonment.

**Conclusions:**

Within the constraints of Brazil’s public health system, affective bonds emerge as an indispensable tool in psychosis care. Consistent with Nise da Silveira’s perspective, therapeutic relationships grounded in empathy and trust may act as stabilizing forces, enabling adherence and improving outcomes where financial and structural resources are limited. These findings highlight the relevance of integrating the affective dimension into psychiatric practice, particularly in contexts of socioeconomic vulnerability.

**Disclosure of Interest:**

None Declared

